# Proteases in Pemphigoid Diseases

**DOI:** 10.3389/fimmu.2019.01454

**Published:** 2019-06-26

**Authors:** Sho Hiroyasu, Christopher T. Turner, Katlyn C. Richardson, David J. Granville

**Affiliations:** ^1^International Collaboration On Repair Discoveries (ICORD), Vancouver Coastal Health Research Institute (VCHRI), Vancouver, BC, Canada; ^2^Department of Pathology and Laboratory Medicine, University of British Columbia (UBC), Vancouver, BC, Canada; ^3^BC Professional Firefighters' Burn and Wound Healing Group, Vancouver Coastal Health Research Institute (VCHRI), University of British Columbia (UBC), Vancouver, BC, Canada

**Keywords:** pemphigoid diseases, proteases, bullous pemphigoid, epidermolysis bullosa acquisita, mucous membrane pemphigoid, elastase, MMP, granzyme

## Abstract

Pemphigoid diseases are a subgroup of autoimmune skin diseases characterized by widespread tense blisters. Standard of care typically involves immunosuppressive treatments, which may be insufficient and are often associated with significant adverse events. As such, a deeper understanding of the pathomechanism(s) of pemphigoid diseases is necessary in order to identify improved therapeutic approaches. A major initiator of pemphigoid diseases is the accumulation of autoantibodies against proteins at the dermal-epidermal junction (DEJ), followed by protease activation at the lesion. The contribution of proteases to pemphigoid disease pathogenesis has been investigated using a combination of *in vitro* and *in vivo* models. These studies suggest proteolytic degradation of anchoring proteins proximal to the DEJ is crucial for dermal-epidermal separation and blister formation. In addition, proteases can also augment inflammation, expose autoantigenic cryptic epitopes, and/or provoke autoantigen spreading, which are all important in pemphigoid disease pathology. The present review summarizes and critically evaluates the current understanding with respect to the role of proteases in pemphigoid diseases.

## Characteristics of Pemphigoid Diseases

The term pemphigoid disease is defined as a specific subset of autoimmune subepidermal blistering diseases having autoantibodies against proteins at the dermal epidermal junction (DEJ) (1). This group includes bullous pemphigoid (BP), epidermolysis bullosa acquisita (EBA), pemphigoid gestationis (PG), mucous membrane pemphigoid (MMPh), linear IgA bullous dermatosis also known as linear IgA disease (LABD), anti-laminin γ1 pemphigoid, lichen planus pemphigoid (LPP), and other rare diseases. Dermatitis herpetiformis (DH) is not included since its autoantigen (transglutaminase) does not localize at the DEJ (1, 2).

Pemphigoid diseases typically share a similar clinical presentation as either localized or generalized tense blisters and erosion on the skin (1). However, this presentation varies for each disease and there is heterogeneity within the same disease. BP typically presents as generalized blistering eruptions accompanied/preceded by erythema and pruritis (3). Although the presentation of PG is similar to that of BP, it normally develops during the second trimester of pregnancy (4, 5). The clinical features of EBA are also often similar to that of BP (referred to as an inflammatory variant of EBA), however, one third of the patients exhibit less inflammation (classical mechanobullous variant) (6–8). In MMPh, blistering and erosive lesions preferably but not exclusively develop on mucosa, such as the oral cavity and conjunctiva, genitalia, perianal region, pharynx, esophagus, and nasal (9, 10). This may result in critical complications such as blindness and strictures. Unlike other pemphigoid diseases, EBA and MMPh lesions may heal with scarring and/or milia formation (6, 9). LABD presents with generalized tense blisters with eruption characteristically accompanied by pruritus (11).

Histology of blistered skin in pemphigoid diseases normally shows superficial and mid-dermis perivascular inflammation infiltrated by lymphocytes, neutrophils, eosinophils, mast cells, and other immune cells, with the relative abundance and contribution depending on each disease (1, 12). The hallmark of BP lesions is eosinophil infiltration, whilst MMPh and classical variant EBA lesions exhibit minimal inflammation compared to the other pemphigoid diseases (12–14). Direct immunofluorescent microscopy of the patient skin is used diagnostically to visualize the deposition of immunoglobulins and/or complements along the basement membrane zone (1, 9, 11). Further analysis with direct (using patient prelesional skin) and/or indirect (using healthy human skin treated with patient serum) immunofluorescent microscopy of skin treated with 1M NaCl solution (salt-split skin) is sometimes clinically used to test the localization of the immunoglobulins (15, 16). Since this salt-split treatment separates the skin at the level of lamina lucida and the localizations of target autoantigens in each disease are characteristic [detailed in the Pathomechanism(s) of Pemphigoid Diseases section], salt-split skins of BP, PG, LPP, LABD, and most of MMPh show the immunoglobulin deposition in the epidermal side or in both the epidermal and dermal sides. On the other hand, the deposition of immunoglobulins is observed in the dermal side of the salt-split skin in EBA, anti-laminin γ1 pemphigoid, and a subset of MMPh (16–18). To more specifically differentiate between the pemphigoid diseases, identification of target antigens for the autoantibodies is required, using enzyme-linked immunosorbent assay (ELISA) and/or western blotting (19–21).

The combined prevalence of pemphigoid diseases was estimated at 380 cases per million people (pmp) (22). BP, the most common disease within this group, was estimated at 259 pmp. The affected population in BP is increasing over time, presumably linked to the increasing risk factors such as aging, pharmacologics, and improved diagnostic techniques (1, 16, 23). In MMPh, prevalence was up to 25 pmp, whilst in EBA it was estimated at about 3 pmp. Other pemphigoid diseases including LABD and anti-laminin γ1 pemphigoid were estimated as 5 pmp. PG in expectant mothers was diagnosed in approximately 1 out of 1,700–50,000 pregnancies (4). While BP and MMPh onset occurs typically in the elderly (median age of onset is ~80 and 70 years, respectively), other pemphigoid diseases show different age distributions (22). Onset of EBA is typically in the elderly (20% of patients are over 70 years old), although a second onset peak has been identified in individuals younger than 30 years old (22, 24). LABD onset peaks before the age of 5 and again after 60 years old (25). The mean age of onset in LPP is between 40 and 50 years of age (1), whilst in PG, as the disease develops during pregnancy, the median age of the onset is ~30 years of age (22).

Multiple factors have been reported to trigger pemphigoid disease onset. For BP, several inflammatory skin conditions (such as trauma, burn, ultraviolet irradiation, radiation, surgical wound, ostomy, and skin graft), specific drugs [including aldosterone antagonists, neuroleptics, spironolactone, phenothiazines with aliphatic side chain, loop diuretics, and dipeptidyl peptidase-4 inhibitor (DPP-4i)], vaccination, and viral infection have been indicated to trigger onset (3, 26–34). The association between BP and neurologic diseases such as stroke, epilepsy, Parkinson's disease, multiple sclerosis, dementia, and unipolar or bipolar disorder is well-documented (3, 28, 35). In LABD, skin trauma and exposure to drugs such as vancomycin have been reported as the triggering factors (11, 36, 37). In addition, several case reports have suggested drugs and inflammatory diseases as initiating other pemphigoid diseases (38–41).

Current treatment modalities for pemphigoid diseases mainly non-specifically target the inflammatory response as their main treatment options, corticosteroids, and immunosuppressive drugs target both innate and adaptive immunities (42–44). For BP, systemic corticosteroid administration remains the standard treatment, however, higher doses of prednisolone may cause critical adverse effects such as diabetes, decreased bone density, and increased susceptibility to infection (45, 46). Topical application of high potency corticosteroids is also used in clinical practice (16, 46, 47). Dapsone, a sulfone with antibacterial properties that is responsible for controlling neutrophil-induced inflammation in the skin, may be used in combination with topical/systemic corticosteroids (48). Other treatment options include systemic administration of a combination of nicotinamide and tetracyclines (tetracycline, doxycycline, or minocycline) (49, 50). Adjuvant immunosuppression with either mycophenolate mofetil or azathioprine has been reported (51). Rituximab, intravenous immunoglobulin, omalizumab, and immunoadsorption have been also reported to show positive effect on the disease course (52–55). PG treatment basically follows a similar course to that of BP including topical corticosteroids and/or low dose systemic corticosteroids (4, 47). LABD often responds well with dapsone (47). EBA is normally treated with systemic corticosteroids in combination with other immunosuppressive/modulatory agents (24). While mild cases of MMPh are often treated with dapsone, severe cases with critical mucosal complications are treated with more aggressive immunosuppressive treatments such as pulse intravenous corticosteroids, cyclophosphamide, or rituximab (9). Overall, pemphigoid disease treatment remains non-specific and often with critical adverse effects. As such, a deeper understanding of the pathology of these diseases is necessary to identify more specific and safer therapeutic approaches.

## Pathomechanism(s) of Pemphigoid Diseases

The hallmark of pemphigoid diseases is the deposition of autoantibodies targeting specific protein(s) at the DEJ (1). The protein or combination of proteins recognized by the autoantibodies vary for each specific pemphigoid disease: collagen XVII [BP180, bullous pemphigoid antigen 2 (BPAG2)] and/or BPAG1e (BP230, dystonin) for BP, PG, and LPP, collagen VII for EBA, collagen XVII, BPAG1e, laminin-332 (laminin-5), laminin-311 (laminin-6), collagen VII, or β4 integrin for MMPh, truncated collagen XVII fragments [linear IgA disease antigen-1 (LAD-1), linear IgA bullous disease antigen of 97 kDa (LABD97)], and/or BPAG1e for LABD, and laminin γ1 for anti-laminin γ1 pemphigoid (56–74). Most of these autoantigens are components or associated proteins of a DEJ anchoring complex, hemidesmosome. Hemidesmosomes are expressed by basal epithelial cells and perform an anchoring function in the skin between the epidermis and dermis (75, 76). In skin, the hemidesmosome consists of transmembrane proteins such as α6β4integrin, collagen XVII, and CD151, and cytoplasmic proteins such as BPAG1e and plectin, to link cytoplasmic keratin with extracellular laminin-332. Laminin-332 binds to collagen VII in the anchoring fibrils. Saliently, genetic mutations of these proteins cause congenital blistering diseases (i.e., epidermolysis bullosa) (77).

It remains unclear as to how immune tolerance is lost in pemphigoid diseases and how/why autoantibodies are formed against hemidesmosome-associated proteins. Several genetical and/or environmental backgrounds, such as human leukocyte antigen (HLA) allele and regulatory T cell dysfunction were suggested to increase autoreactive T and B cells in the pemphigoid diseases (78–85). These autoreactive lymphocytes possibly react with hemidesmosome-associated protein fragments disseminated in the extracellular space by exaggerated proteolytic cleavages at the DEJ during the aforementioned triggering events including skin inflammatory diseases and immunization.

The pathological functions of autoantibodies in blister formation has been studied using passive transfer mouse models. The models involve injections of anti-mouse collagen XVII IgG, anti-mouse collagen VII IgG, anti-laminin-332 IgG, or anti-human LAD-1/LABD97 IgA into healthy wild-type or human skin transplanted mice, resulting in the development of BP, inflammatory variants of EBA, anti-laminin-332 MMPh, or LABD model, respectively (86–91). Most of these animal models demonstrate the deposition of immunoglobulin and complements C3 at the DEJ, infiltration of inflammatory cells, and the presentation of subepidermal blistering. *Ex vivo* skin systems also provide a valuable research tool to reveal pemphigoid disease pathology (92). Cryosections of healthy skin are incubated with patient-derived IgG and leukocytes, leading to the induction of dermal-epidermal separation (93, 94). Based on these studies, it is now recognized that the blisters present in most pemphigoid diseases are triggered by the accumulation of autoantibodies at the DEJ followed by complement recruitment and inflammatory cell infiltration.

Passive-transfer mouse models of MMPh developed by Lazarova et al. and Darling et al. showed subepidermal blisters with IgG and C3 deposition but without obvious inflammation (90, 91). In addition, in one *ex vivo* skin study with anti-laminin-332 MMPh patient IgG, there was a failure to induce leukocyte recruitment and dermal-epidermal separation, suggesting an inflammation-independent mechanism is involved in blister formation in laminin-332 MMPh (19, 95). Conversely, a recent study using the anti-laminin-332 MMPh model developed by Heppe et al. showed complement activation and inflammation are indeed required for blister formation (88). Further studies are therefore needed to further elucidate the mechanisms in anti-laminin-332 MMPh.

*Ex vivo* skin- and passive transfer murine-models of pemphigoid diseases have demonstrated that neutrophils are especially important amongst the infiltrated inflammatory cells in blister formation (93, 94, 96). The *ex vivo* skin model showed neutrophils to be indispensable for BP and EBA blister formation as the patient IgG induced dermal-epidermal separations were only observed when co-incubated with neutrophils (93, 94). Liu et al. utilized the passive-transfer mouse model to demonstrate the importance of neutrophils in BP pathology, as depletion of circulating neutrophils in the BP mice showed resistance to blistering (96). To fight against pathogens, neutrophils provide reactive oxygen species (ROS), antimicrobial peptides, and proteases (97, 98). Since blister formation should be induced by the loss of epidermis and dermis attachment, it validated subsequent studies focusing on the function of proteases on the cleavage of anchoring proteins at the DEJ, such as hemidesmosomal components.

## Proteases in Pemphigoid Diseases

Proteases are classically categorized into six groups based on the catalytic residue; serine, cysteine, aspartic, glutamic, threonine, and metalloproteases (99). Proteases exert both physiological and pathological roles through proteolytic cleavage and degradation of wide variety of substrates such as extracellular matrices, cell surface molecules, transmembrane proteins, growth factors, cytokines, and chemokines. The remainder of this review will summarize the current understanding with respect to the role of proteases in the pathogenesis of pemphigoid diseases.

### Neutrophil Elastase

Neutrophil elastase (NE) is a serine protease that exhibits relatively broad cleavage site specificity and has a preference for regions containing several aliphatic amino acids (100). NE is stored in both azurophilic (also called primary) granules and the nuclear envelop of neutrophils as an active-form (101–103). Following bacterial infection and subsequent inflammatory stimulation, neutrophils phagocytose the invading bacteria, with NE contributing to intracellular killing (104, 105). In addition, upon neutrophil activation, NE is also secreted into the extracellular space, acting anti-bacterially to degrade bacterial proteins and various virulence factors such as outer membrane protein, flagellin, and leukotoxin (101, 106–108). NE also cleaves targets within the skin such as chemokines, cytokines, growth factors, cell surface molecules, adhesion proteins, and extracellular matrices (101, 109–113). These proteolytic functions serve to augment inflammation and to repair tissue at early phases of wound healing. However, excessive NE activity may cause unintended pathological consequences. Exaggerated NE-mediated proteolysis has been implicated as a key factor in inflammatory diseases [chronic obstructive pulmonary disease (COPD), cystic fibrosis, acute lung injury, acute respiratory distress syndrome], autoimmune diseases (type 1 diabetes), cancer (squamous cell carcinoma), and inflammatory skin diseases (psoriasis, skin photoaging) (101, 114–120). To defend against excessive NE proteolysis, there are endogenous secretory NE inhibitors such as α1-antitrypsin (α1-AT), serpin B1, proteinase inhibitor-9 (PI-9, serpinB9), chelonianin, and macroglobulin (114). However, an imbalance of local protease-antiprotease activity has been observed, likely due to genetics, environmental factors, or simply an inability to cope with the massive degree of inflammation (101, 120, 121). In this context, the function of NE in pathology and underlying pemphigoid diseases remains a topic of further study.

**Figure 1:**
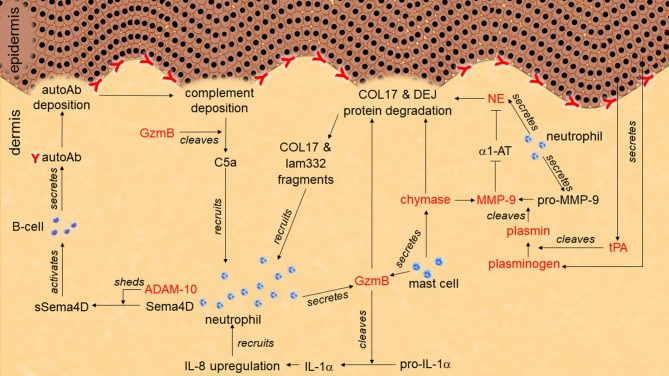
Role of proteases in pemphigoid disease. Tissue-type plasminogen activator (tPA) secreted from keratinocytes activates plasminogen to plasmin. Plasmin activates pro-matrix metalloprotease-9 (pro-MMP-9) to MMP-9. MMP-9 degrades α1-antitrypsin (α1-AT). Without inhibition by α1-AT, neutrophil elastase (NE) cleaves hemidesmosome-associated proteins including collagen XVII (COL17) and laminin-332 (lam332). Granzyme B (GzmB) also cleaves hemidesmosome-associating proteins to induce dermal-epidermal separation. GzmB may induce additional neutrophil infiltration through chemoattractant production such as IL-1α, C5a, and COL17/lam332 fragments. ADAM-10 sheds semaphorin-4D (sema4D) to activate autoantibodies (autoAb) production from B cells.

Abundant NE-positive neutrophils and NE activity have been reported in human BP blister fluid (122–124) (Table 1). A direct link between NE and blistering was identified using the passive-transfer BP model with anti-mouse collagen XVII IgG where NE null mutant mice or wild type mice administered NE inhibitors (α1-AT and MeOSuc-AAPV-CH_2_Cl) were resistant to blister formation (125, 126). In addition, in the *ex vivo* human skin model, leukocytes and BP patient IgG dependent dermal-epidermal separation was blocked with a NE inhibitor (MeOSuc-AAPV-CK) (95). Using the same model but with IgG from EBA patients, it was confirmed that pathogenic IgG in EBA patients also contributes to NE-dependent blister formation (95). NE-induced blistering in BP and EBA was proposed to be generated by the degradation of hemidesmosomal proteins including collagen XVII (126, 127) (Figure 1; Table 1). NE also cleaved laminin-332 *in vitro*, which is another hemidesmosome-associated protein (128).

**Table 1:** Major proteases in pemphigoid diseases.

**Protease**	**Class**	**Location in PD**	**Functions in PD**	**References**
NE	serine protease	BP—blister fluids	BP, EBA Hemidesmosomal protein degradation (COL17 and laminin-332)	(95, 122–127)
MMP-2 (gelatinase A, 72 kDa type IV collagenase)	Metalloprotease	BP—blister fluids, lesional skin EBA—sera MMPh—tear, sera	EBA Complexed with Hsp-90	(124, 156, 165, 166)
MMP-3 (stromelysin-1)	Metalloprotease	BP—serum, lesional skin	Negative in BP mouse model	(163, 167, 168)
MMP-9 (gelatinase B, 92 kDa type IV collagenase)	Metalloprotease	BP—lesional and perilesional skin, blister fluid MMPh—tear	BP, EBA NE activation through α1-AT degradation CD46 shedding	(96, 124, 125, 148, 155–160, 163, 164)
MMP-12 (macrophage elastase)	Metalloprotease	EBA—sera BP—lesional skin	EBA Complexed with Hsp-90	(166, 169)
MMP-13 (collagenase-3)	Metalloprotease	BP—lesional skin	Unknown	(156)
Plasmin, plasminogen, tPA, and uPA	Serine protease	BP—blister fluid, lesional skin	BP MMP-9 activation COL17 shedding	(163, 201–207)
Chymase/mMCP-4	Serine protease	BP—non lesional skin	BP MMP-9 activation COL17 degradation	(244, 304)
Granzyme B	Serine protease	BP, EBA, DH—lesional skin	DEJ protein degradation (COL7, α6β4 integrins, COL17) IL-1α activation C5a production	(298, 299)
ADAM-8	Metalloprotease	BP—epidermis of lesional skin	Unknown	(159, 258, 259)
ADAM-9	Metalloprotease	BP—epidermis of lesional skin	COL17 shedding	(159, 258, 259)
ADAM-10	Metalloprotease	BP—epidermis of lesional skin	COL17 shedding CD46 shedding Semaphorin 4D shedding	(159, 164, 258, 259)
ADAM-15	Metalloprotease	BP—epidermis of lesional skin	Unknown	(159, 258, 259)
ADAM-17	Metalloprotease	BP—epidermis of lesional skin	(Indirect) COL17 shedding	(159, 258, 259)
Tryptase	Serine protease	BP—blister fluid, sera	Unknown	(302–304)
Cathepsin-G	Serine protease	BP	Negative in BP mouse model	(126, 163, 301)

The degradation of hemidesmosomal proteins might exaggerate the inflammatory response in pemphigoid disease. Mydel et al. and Lin et al. indicated that NE-induced fragments of laminin-332 and collagen XVII are chemotactic for neutrophils (127, 128). Bergh et al. demonstrated that loss of collagen XVII induces IL-8 expression in keratinocytes, which potentially induces further inflammation in BP (129).

Based on its role in pemphigoid diseases, NE has been proposed as a therapeutic target. However, there has been no reported clinical evidence forwarded that supports the use of NE-inhibiting drugs such as sivelestat (ONO-5046) or AZD9668 for pemphigoid diseases (130, 131). One recent paper suggested a possible mechanism which may induce resistance against macromolecular NE inhibitors (132). It was proposed that the closed compartment between neutrophils and immune complexes prohibits the access of inhibitors, which implies NE inhibition as a treatment strategy for pemphigoid diseases may be challenging.

### Matrix Metalloproteases (MMPs)

MMPs (also known as matrixins) are a family of calcium-dependent zinc-containing proteases generally consisting of a signaling peptide-, propeptide-, catalytic-, and hemopexin-like-domains (133, 134). To activate these proteolytic functions, the interaction between catalytic domain and propeptide domain needs to be removed normally by other proteases, such as plasmin, trypsin, kallikrein, tryptase, and other MMPs (134–137). Once activated, MMPs are available to cleave a diverse range of substrates such as chemokines, cytokines, growth factors, cell surface molecules, adhesion proteins, extracellular matrices, and other proteases (134, 138). Because of this wide range of substrates, MMPs play a number of roles in physiological processes, including in inflammatory responses, angiogenesis, reproduction, development, wound closure, and tissue remodeling (133, 134, 139–142). To avoid excess host tissue damage and unregulated inflammation, endogenous inhibitors such as α2-macroglobulin and all types of tissue inhibitor of matrix metalloproteinases (TIMPs) block excessive enzymatic activity of MMPs (137, 143). However, and similar to NE, several reasons may create an imbalance between proteases and antiproteases, resulting in multiple diseases. MMPs have been implicated in pathological roles in cancer, inflammatory diseases, autoimmune diseases, neuropsychiatric disorders, central nervous system diseases, cardiovascular diseases, and delayed wound healing (134, 137, 139, 142, 144–146). The pathological functions of MMPs in pemphigoid diseases have been studied, predominantly focusing on MMP-9.

MMP-9, also known as gelatinase B or 92 kDa type IV collagenase, is secreted from several cell types including neutrophils, macrophages, eosinophils, and fibroblasts (147, 148). In neutrophils, MMP-9 is stored in zymogen granules and secreted upon an inflammatory stimulation (149, 150). Conversely, in macrophages, MMP-9 does not accumulate and instead is secreted as a 92-kDa proactive form following synthesis (151). Once activated, the 88-kDa active form of MMP-9 extracellularly cleaves a variety of substrates such as chemokines, cytokines, growth factors, cell surface molecules, transmembrane proteins, extracellular matrices, and proteases (147, 152–154).

While multiple studies report MMP-9 positive keratinocytes, neutrophils, T-cells, mast cells, and eosinophils to be abundant in lesional and perilesional BP skin (Table 1), Verraes et al. indicated that blister fluid MMP-9 may present only as proenzyme and therefore not able to degrade collagen XVII (124, 148, 155–159). Moreover, they indicated that TIMP-1 is abundant in the blister fluids, which would likely inhibit activity of MMP-9. On the other hand, Niimi et al. suggested TIMP-1 expression was less compared to MMP-9 at the BP lesion (156). In MMPh, MMP-9 protein levels and the MMP-9/TIMP-1 ratio were increased in patient tears (160, 161) (Table 1).

Once activated, and in the absence of inhibition, MMP-9 degrades the extracellular domain of human collagen XVII and the NE inhibitor, α1-AT (124, 125). The role of MMP-9 in BP and EBA blistering was confirmed with *ex vivo* human skin models (95). Cryosections of human skin incubated with BP- or EBA-patient IgG and leukocytes created dermal-epidermal separation through an MMP-9-dependent manner as it was blocked by the MMP-9 inhibitor, 3G12scFV. Passive transfer BP mice showed MMP-9 activation at the lesional skin, whilst MMP-9 deficiency induced resistance to blister formation (162, 163). MMP-9 is likely to induce blistering through NE activation by degrading α1-AT, but not through direct-collagen XVII degradation, as direct stimulation with MMP-9 did not induce dermal-epidermal separation in *ex vivo* mouse skin sections (125) (Figure 1; Table 1). MMP-9 has also been indicated as having a role in complement activation in BP through CD46 shedding (164).

Other than MMP-9, MMP-2, -3, -12, and -13 have been reported to be upregulated in pemphigoid diseases (124, 156, 165–169). MMP-2 (gelatinase A, type IV collagenase) is ubiquitously and constitutively expressed in many cells and tissues including dermal fibroblasts (170). Multiple physiological and pathological roles have been indicated for MMP-2 in angiogenesis, tissue repair, cancer, and inflammation through the cleavage on cytokines, chemokines, cell surface proteins, extracellular matrices, and proMMPs. MMP-2 has been identified in the tears and sera of MMPh patient, blister fluids and lesional skins of BP, and sera of EBA (124, 156, 165, 166) (Table 1). MMP-2 is predicted to regulate Hsp-90-dependent blister formation through ROS release in EBA, since MMP-2 is complexed with Hsp-90 to be stabilized by the chaperone in the patient sera (166) (Table 1). MMP-2 cleaves some anchoring proteins such as collagen XVII, collagen VII, and laminin-332 *in vitro*, however, the direct function in the pathology remains to be elucidated (171, 172).

MMP-3 (stromelysin-1) exhibits multiple functions in development, inflammation, cancer, wound repair, skin inflammation through proteolyses on cytokines, chemokines, cell surface proteins, extracellular matrices, growth factors, proMMPs, and protease inhibitors (134, 170, 173). Increased MMP-3 has been detected in BP serum and lesional skin (167, 168) (Table 1). *In vitro*, MMP-3 can activate MMP-9 (174). However, MMP-3 deficient mice fail to display impaired MMP-9 activation and were still susceptible to experimental BP, suggesting that MMP-3 is dispensable to the pathology of BP (163) (Table 1).

MMP-12 (macrophage elastase) is produced in and secreted from mainly macrophages but also detected in other cell types including dermal fibroblast and vascular smooth muscle cells (170, 175). Through the proteolysis of cytokines, chemokines, cell surface proteins, extracellular matrices, proteases, and bacterial cellular membranes, MMP-12 contributes to inflammation, infection, tissue remodeling, and cancer. Increased MMP-12 has been observed in EBA sera and the lesional skin of BP (166, 169) (Table 1). In the EBA patient sera, and the same as observed for MMP-2, MMP-12 is complexed with Hsp-90 to regulate Hsp-90-dependent blister formation through ROS release (166) (Table 1). The direct function of MMP-12 in pemphigoid diseases remains unknown, however, MMP-12 cleaves laminin-332, suggesting it may directly cause dermal-epidermal separation (128).

MMP-13 (collagenase-3) is distributed in multiple cell types such as in connective tissue, epithelial cells, and neural cells (134, 170). It cleaves cytokines, chemokines, extracellular matrices, proMMPs, and protease inhibitor to exhibit functions in inflammation, cancer, and tissue remodeling. Increased MMP-13 positive cells have been detected in lesional skin of BP (156) (Table 1). Although its role in pemphigoid diseases has not been studied, it may contribute to disease through MMP-9 activation, which have been indicated before (176).

As mentioned above, a number of studies implicate MMPs (especially MMP-9) as promising targets for pemphigoid disease treatment. However, it should be noted that therapeutic use of broad spectrum MMP inhibitors have failed in cancer clinical trials with a lack of efficacy and adverse effects possibly caused by inhibiting the essential physiological roles of MMPs (139). Indeed, multiple MMPs appear to exert beneficial functions such as anti-tumorigenesis and/or anti-inflammation and have therefore been proposed as “anti-targets” whereby their inhibitions would cause severe adverse effects (139). For example, since MMP-9 also exhibits aforementioned critical physiological roles, it is not surprising that even the specific MMP-9 inhibitor, andecaliximab showed several adverse effects in the clinical trial, such as nausea, vomiting, fatigue, diarrhea, asthenia, arthralgia, joint stiffness, and dyspnea, which would not be tolerated in treatments for benign diseases such as pemphigoid diseases (177). There are no reports of MMP inhibitors such as andecaliximab being tried as a therapeutic approach to treat pemphigoid diseases. Notably, doxycycline has been reported to regulate MMP-9 activation in other organs (178–181). Although its mechanism in the BP treatment is still unclear, Williams et al. reported 200 mg/day oral doxycycline is as effective as 0.5 mg/kg/day oral prednisolone (50).

### Plasmin, Plasminogen, Tissue-Type Plasminogen Activator (tPA), and Urokinase-Type Plasminogen Activator (uPA)

Plasmin is a serine protease well-recognized as functioning in the fibrinolytic cascade (182, 183). Its precursor, plasminogen is created in liver cells and secreted into plasma (184, 185). Subsequently, plasminogen is cleaved by tPA and uPA to generate plasmin. Plasmin preferably cleaves following the arginine or lysine residues (186). As an important factor in the fibrinolytic system, plasmin degrades fibrin clots, thus prevents pathological conditions such as thrombosis (183). In addition to fibrin, plasmin cleaves many other substrates including coagulation factors, complement C3 and C5, hormones, metalloproteases, growth factors, cytokines, chemokines, cell surface molecules, and extracellular matrices (184, 187–194). With this variety of cleavage substrates, plasmin has been linked to multiple physiological processes such as inflammation, wound healing, and tissue remodeling (182, 195, 196). To prevent excessive proteolysis, plasmin activity is regulated by endogenous inhibitor, α2-antiplasmin (184). However, and similar to other proteases, an imbalance between plasmin and its inhibitor trigger pathological conditions, for example in cancer and inflammatory diseases (inflammatory response after the major surgery and trauma, asthma, COPD, and central nervous system inflammation) (182, 197–200).

Elevated levels of active plasmin and tPA are present in blister fluid and the lesional skin of BP patients (201–206) (Table 1). Keratinocytes stimulated by BP-patient IgG release tPA (202) (Figure 1). The function of the plasminogen/plasmin system in this context was confirmed using the passive-transfer BP model, where the administration of a plasmin inhibitor (α2-antiplasmin) blocked blistering (163). Mice deficient of plasminogen, and both tPA and uPA exhibit delayed and less intense blistering in the passive-transfer BP model. Since all of these deficient mice reconstituted BP with active MMP-9 but not with the proMMP-9, the PA/plasminogen/plasmin cascade is likely to induce blistering through MMP-9 activation (Figure 1; Table 1).

Intriguingly, Hofmann et al. demonstrated using *in vitro* system that plasmin generates 97-kDa fragments of collagen XVII known as LABD97 (203). Similarly, Nishie et al. showed that BP blister fluid cleaves recombinant collagen XVII into 120-kDa ectodomain in a plasmin-dependent manner (207). They suggested that this plasmin-induced cleavage of NC16a domain in collagen XVII generates neoepitopes possibly involved in the onset of BP and LABD (Table 1). As a related topic, Izumi et al. suggested that plasmin inhibition with DPP-4i induced characteristic non-inflammatory BP, possibly through plasmin independent collagen XVII cleavage, and the generation of neoepitopes within different domains by other proteases (208). The physiological role of collagen XVII shedding in re-epithelialization was indicated using a non-shedding collagen XVII mouse model, which exclusively expresses non-sheddable collagen XVII mutant (209).

Anti-plasmin drugs such as ε-aminocaproic acid and tranexamic acid are mostly used to inhibit fibrinolysis (182). Intriguingly, Grando has reported the pemphigoid disease treatment using a combination of oral prednisolone, ε-aminocaproic acid, and aprotinin, which is an inhibitor of serine proteases including plasmin (210). However, the therapeutic effect of this treatment approach compared to the control group (prednisolone alone) has not been reported.

### Chymase and Mouse Mast Cell Protease 4 (mMCP-4)

In human tissues, infiltrating and degranulating mast cells were associated with BP (211). The importance of mast cells in the pathology of BP has been suggested, in part through the use of the passive-transfer model with anti-mouse collagen XVII IgG on Kit or Scf [stem cell factor, Kitl (kit ligand)]-mutation dependent mast cell-deficient mice, which failed to develop BP (212). Since intradermal injection of either polymorphonuclear leukocytes or IL-8 (a neutrophil chemoattractant) recovered the lack of phenotypes on Kit- or Scf-mutation mice, they concluded that mast cells play an essential role in neutrophil recruitment in BP. However, as recent studies revealed that Kit- or Scf-mutation affects not only mast cells but also multiple cell types including those of immune- and non-immune origin, this result may be questioned (213, 214). The recently developed Kit- or Scf-mutant independent mast cell deficient mice should be tested for further analysis. It should be also noted that blocking mast cell degranulation with the inhibitor (cromolyn sodium) in BP mice significantly reduced disease phenotype as well, thereby indicating the importance of mast cell granules in BP pathogenesis (212, 215).

Although often believed that the pathological mechanisms operating in BP and inflammatory variant EBA are quite similar, at least in the passive-transfer disease models, mast cells may participate differently in each. Both Kit mutation-dependent and -independent mast cell deficiencies induced consistent blistering phenotypes in passive-transfer mouse model of EBA, even though activated mast cells were abundant in the lesions of the EBA in wild-type mice (216). The results indicate that, in contrast to the BP model, mast cells and secreted proteases appear to be dispensable for the blister formation in EBA.

Human mast cells release proteases including chymase, tryptase, cathepsin G, carboxypeptidase A3, dipeptidtlpeptidase I/cathepsin C, cathepsins L and S, granzyme B, plasminogen activators, and MMPs (217). One of the major granule components of mast cells, chymase, is a serine protease that cleaves peptides after aromatic amino acids, preferably phenylalanine and tyrosine residues (217, 218). It is produced as an inactive form in mast cells and activated by cleavage with dipeptidyl peptidase I (DPPI) within the granules (219). Following stimulation, such as during inflammation or injury, chymase is released into the extracellular space. Chymase is resistant to multiple endogenous inhibitors such as α1-AT, α2-antichymotrypsin, α2-macroglobulin, and eglin C, when bound to heparin proteoglycan (220). While chymase is well-recognized for its ability to convert angiotensin I to its active form, angiotensin II, it also reportedly cleaves cytokines, growth factors, proteases, transmembrane proteins, and extracellular matrices (221–233). Although rodents have multiple isoforms of chymase, mMCP-4 is recognized as the isoform comparable to human chymase because of its biophysical and functional properties and tissue distribution (221). Based on former studies using deficient mice in this functional-equivalent, chymase has been revealed to function in the regulation of inflammatory response and tissue remodeling (221, 234, 235). Chymase has also been suggested to exert pathological roles in multiple diseases such as cancers, cardiovascular diseases, inflammatory lung diseases (idiopathic pulmonary fibrosis, COPD), renal diseases (diabetic nephropathy, hypertensive nephropathy, rejected kidney allograft), preeclampsia, skin keloid, and atopic dermatitis (220, 221, 236–243).

The critical contribution of mMCP-4 in disease mechanisms is also observed in BP as passive-transfer mouse model with anti-mouse collagen XVII IgG on mMCP-4 deficient mice showed resistant to blistering even neutrophil recruitment was observed (244). Since impaired activation of MMP-9 in the mMCP-4 deficient BP mice and degradation of collagen XVII by mMCP-4 *in vitro* were observed, they indicated that mMCP-4 affects BP pathology by both activating MMP-9 and degrading collagen XVII (Figure 1; Table 1). On the other hand, cathepsin G/chymase inhibitors (α1-antichymotrypsin or Z-Gly-Leu-Phe-CH_2_Cl) did not improve passive-transfer BP model (126).

A few chymase inhibitors were and are being tested in phase II clinical trials for heart failure, diabetic kidney disease, or atopic dermatitis (237). The trial of SUN13834 on atopic dermatitis was discontinued because of adverse side effects. So far, there is no report of chymase inhibitors being tested on pemphigoid disease patients.

### A Disintegrin and Metalloproteases (ADAMs)

ADAMs are a family of single-pass transmembrane proteins consisting of an extracellular metalloprotease domain, a disintegrin domain, a cysteine rich domain, a transmembrane domain, and a cytoplasmic tail (245). Although all members of ADAMs contain metalloprotease domains, some of them do not possess functional protease activity. Only ADAMs 8, 9, 10, 12, 15, 17, 19, 20, 21, 28, and 33 are recognized as exhibiting proteolytic activity, requiring removal of the extracellular end prodomain within the cytoplasm (246). These functional ADAM metalloproteases mainly regulate ectodomain shedding on multiple cell surface proteins, which results in regulation of growth factors, cytokines, chemokines, adhesion molecules, and receptors in order to control physiological systems such as inflammation and development (247, 248). These proteolytic activities are controlled by endogenous inhibitors such as TIMPs and by their cellular localizations regulated by endocytosis (249–251). However, as with other proteases, dysregulation is often observed in several diseases. Pathological roles of ADAMs have now been reported in cancers, wound healing, psoriasis, rheumatoid arthritis, inflammatory lung diseases, inflammatory bowel diseases, predominantly functioning through ectodomain shedding of cytokines, chemokines, and chemoattractant (247, 252–257).

In BP, elevated protein levels of ADAMs 8, 9, 10, 15, and 17 in the epidermis of the lesional skins have been indicated (159, 258, 259) (Table 1). ADAMs 9, 10, and 17 are regulated by TWEAK/Fn14 pathway and may participate in collagen XVII loss in the skin lesion of BP (159). Upregulated ADAM10 has also been suggested to shed CD46, which results in enhancement of complement activation in BP lesions (164). Moreover, ADAM10 sheds soluble semaphorin 4D from the granulocytes to activate B cells, which results in enhancing autoantibody production in BP (259) (Figure 1; Table 1).

Intriguingly enough, mainly ADAMs 9 and 10, but also indirectly ADAM17, constitutively shed 120-kDa ectodomain of collagen XVII, LAD-1 (260, 261) (Table 1). ADAMs may also play a role in neoepitope production through collagen XVII cleavage, possibly triggering BP and LABD onset (207).

Inhibitors targeting broad spectrum of ADAMs have failed clinical trials primarily due to adverse effects (262). Development of drugs that target specific ADAM is challenging due to structural similarities in ADAMs and MMPs. In addition, many substrates of ADAMs are shared with other ADAMs and MMPs. Therefore, specific ADAM inhibitor may not be sufficient to provide good efficacy. A small molecule inhibitor of ADAMs 10 and 17, INCB7839 has been tested in a breast cancer clinical trial, which was discontinued likely because of increased deep vein thrombosis (263). This drug is now being tested in diffuse large B cell non-Hodgkin lymphoma phase II clinical trial. There is no report of using ADAM inhibitors on pemphigoid diseases.

### Granzyme B

Granzymes (Gzms) are a family of serine proteases that includes five members in humans: GzmA, GzmB, GzmH, GzmK, and GzmM (264, 265). Discovered in the granules of cytotoxic T cells and natural killer (NK) cells, granzymes were traditionally considered exclusively as key mediators of granule-induced cell death, targeting cancer or virally infected cells. GzmB initiates apoptosis through caspase-dependent and/or caspase-independent pathways after internalized into target cells (266, 267). For internalization, another granule component, perforin, is required to form pores on the target cell membrane (268, 269). Saliently, not all secreted GzmB is internalized by the target cells as approximately one-third escapes from the immunological synapse and into the extracellular space (270). Moreover, GzmB is secreted by cells not involved in cytotoxicity or perforin release, including immune- (mast cells, neutrophils, macrophages, basophils, dendritic cells, and regulatory T cells) and non-immune (keratinocytes and chondrocytes) cells (271–281). In contrast to other proteases which are tightly regulated in the extracellular spaces, GzmB-mediated proteolysis in the extracellular space is not likely to regulated by the endogenous inhibitors, since the only inhibitor identified thus far in human tissue, PI-9 is located in the cytoplasm and not secreted into the extracellular space (282). Therefore, GzmB is expected to exhibit alternative roles in the extracellular space through its proteolytic activity.

GzmB has cleavage specificity after an aspartic acid or glutamic acid residues (283). Multiple extracellular substrates for GzmB have now been identified *in vitro*, such as cytokines (IL-1α, proIL-18), complements (C3, C5), extracellular proteins (fibronectin, vitronectin, laminin, decorin, biglycan), coagulation/ fibrinolytic factors (von Willebrand factor, plasminogen), and cell surface proteins (VE-cadherin, ZO-1) (284–288). Through these cleavages and degradations in the extracellular spaces, GzmB is expected to regulate inflammation, cell adhesion, cell migration, anoikis, coagulation, fibrinolysis, and cell-cell adhesion. Present at low levels in healthy tissue, GzmB is elevated in numerous pathological conditions such as atherosclerosis, rheumatoid arthritis, transplant rejection, acute graft vs. host disease, discoid lupus, drug eruption, atopic dermatitis, impaired burn wound, and photoaging (279, 289–297). In these diseases, pathological contributions of GzmB are suggested through not only intracellular apoptotic function but also extracellular proteolytic role.

GzmB positive cells localize to blisters in pemphigoid diseases (298, 299) (Table 1). However, since GzmB has long been exclusively recognized as a cytotoxic inducer, the proteolytic role of GzmB in the extracellular space had not been tested in pemphigoid diseases until recently (299). GzmB cleaves multiple anchoring proteins such as α6β4 integrins, collagen VII, and collagen XVII *in vitro*. Moreover, GzmB induces dermal-epidermal separation in *ex vivo* human skin. These results suggest that GzmB-induced cleavage of anchoring proteins directly leads to subepidermal blistering in the pemphigoid diseases (Figure 1; Table 1). Because of its wide variety of substrates in skin and inflammatory conditions, GzmB could exert multiple roles in the pathogenesis of pemphigoid diseases. For example, GzmB proteolytically augments the pro-inflammatory activity of IL-1α, which would be predicted to promote neutrophil accumulation at the lesion through subsequent IL-8 activation (287). In addition, GzmB cleaves C5 to generate a strong chemoattractant, C5a, to cause additional inflammatory cell infiltration (286). Since GzmB directly cleaves collagen XVII, GzmB may also contribute to the neoepitope generation in BP as similar to plasmin and ADAMs. Intriguingly, as GzmB is upregulated with age, it could help to explain its role in age-related autoimmune blistering pathologies such as BP, however further studies are required (3, 300).

Recently, a topical GzmB inhibitor was tested on impaired burn wound murine model, however, there are currently no clinically-approved GzmB inhibitors on the market (289).

### Other Proteases

In addition to the above-mentioned proteases, other proteases have been identified as being upregulated in pemphigoid diseases including tryptase and cathepsin G (163, 301–304). Although the functions of these proteases in pemphigoid diseases remain unclear, we enumerate current understanding of these enzymes in the pemphigoid diseases and relating fields.

Tryptase is a serine protease mainly secreted from mast cells (305, 306). It is well-recognized to activate protease-activated receptor 2 (PAR-2) with its proteolytic activity (307). Through PAR-2 dependent and independent mechanisms, tryptase induces the release of cytokines and chemokines from multiple cell types. Other than PAR-2, it cleaves extracellular matrices and coagulant factors and exhibits a role in inflammation, angiogenesis, anticoagulant, tissue remodeling, cancer, allergic inflammatory diseases, and cardiovascular diseases (305, 306). Tryptase has been identified as being elevated in blister fluids and sera from BP patients (302–304) (Table 1). Protein levels show at least a partial positive correlation with autoantibody titers, cytokines, and clinical severity, however, its function has not been tested in pemphigoid disease models.

Cathepsin G is a serine protease mainly localized in the azurophilic granules of neutrophils (308, 309). With its proteolytic ability on cytokines, chemokines, cell surface proteins, extracellular matrices, outer membrane of infectant, angiotensin II, and proMMPs, cathepsin G exhibit important roles in inflammation, thrombogenesis, host defense, blood pressure, tumor invasion, and autoimmune diseases. Elevated cathepsin G has been observed in BP samples (163, 301) (Table 1). *In vitro* cleavage assays indicated that cathepsin G degrades laminin-332, suggesting it may induce dermal-epidermal separation (128). However, cathepsin G inhibition by α1-antichymotrypsin did not reduce disease severity on passive-transfer mouse model of BP, thus a direct role is yet to be confirmed (126, 163) (Table 1).

Together, further studies are required to fully elucidate the contribution of these proteases to pemphigoid disease pathogenicity.

## Regulators of Proteases in Pemphigoid Diseases

In addition to the above-described regulatory actions by the endogenous inhibitors, proteases are controlled by other multiple factors such as cytokines and different proteases. Since former studies have characterized that the profiles of cytokines and chemokines in pemphigoid diseases are likely to be unique, these characteristic profiles may be important for protease regulation.

Th2 relating cytokines such as IL-4, IL-5, soluble CD30, CCL5 (RANTES), CCL11 (eotaxin), CCL17 (TARC), CCL18 (PARC), CCL22 (MDC), CCL26 (eotaxin 3), and TSLP are elevated in the sera and/or blister fluids of BP patients (310–326). Elevated Th1 cytokines such as IFN-γ, IL-1β, TNF-α, CXCL9 (MIG), CXCL10 (IP10), and IL-18 have been also identified within the BP patient samples (310, 312, 318, 324, 325, 327). Besides them, IL-6, IL-8, IL-17, IL-21, IL-22, and IL-23 are elevated (310, 320, 324, 328, 329). Intriguingly, serum level of IL-17, IL-23, and CXCL10 in follow-up patients were elevated only in patients who later relapsed (328, 330). Since these cytokines and chemokine regulate MMP-9 secretion from inflammatory cells, it has been suggested that elevated IL-17, IL-23, and CXCL10 could trigger relapse through increased MMP-9 secretion (328, 330). Cytokine and chemokine profiles in other pemphigoid diseases are poorly defined at present, presumably due to the rareness of such diseases. Regarding EBA, serum and skin IL-6 expression are increased, however other cytokines did not show a significant increase due to a high degree of variation (331). In the same study, elevated concentration of IL-4, RANTES, IL-1α, IL-1β, TNF-α, IL-6, IL-10, IL-17, MIP-1α, KC, and GM-CSF are detected in the passive-transfer mouse model of EBA. In MMPh, elevated IL-4, IL-5, IL-13, IL-1α, IL-1β, IL-2, IL-12, TNF-α, IL-6, IL-8, IL-17, and TGF-β1 have been detected in serum and/or lesions of the human patients (161, 332–340).

As indicated above as interaction between NE, MMP-9, chymase, and plasmin, the proteases influence each other directly and indirectly by degrading intermediate proteases or protease inhibitors. Identifying the interaction between the proteases in the diseases is challenging since tissues include many types of proteases and each protease has wide variety of substrates. To conquer this conundrum, the field of degradomics was established (341). Combining genomics, proteomics, and bioinformatics, a whole map of complex protease interactions and networks is beginning to be elucidated not only *in vitro*, but also *in vivo* including in diseases such as COPD and pancreatic tumors (154, 342–344). Resulting from these and other studies, proteases have been recognized as influencing the activities of other proteases and, helping to define the “protease web” (342).

## Conclusion

Multiple proteases have been identified as being elevated in pemphigoid diseases. Several have been proposed to play key roles in blistering pathology through the cleavage of hemidesmosomal proteins, resulting in dermal-epidermal separation and blister formation. In addition, some proteases have been suggested to contribute to neoepitope generation and dysregulated inflammatory response in the diseases. Despite significant advancements, further research is required to further elucidate the complex role that proteases play in various pemphigoid diseases.

Inhibition of specific proteases in pemphigoid diseases provides a unique, potentially safer therapeutic approach compared to current non-specific immune suppressive treatments that are often plagued with undesirable adverse effects. Thus far, evidence of clinical efficacy is minimal, but this may change as protease function is further defined, more effective inhibitors are developed, and new trials are commenced.

## Author Contributions

SH, CT, and DG wrote the manuscript. SH and KR prepared the table and figure.

### Conflict of Interest Statement

DG is a co-founder and serves as consultant/Chief Scientific Officer of viDA Therapeutics, Inc. The remaining authors declare that the research was conducted in the absence of any commercial or financial relationships that could be construed as a potential conflict of interest.

## Abbreviations

α1-AT: α1-antitrypsin
ADAM: a disintegrin and metalloprotease
BP: bullous pemphigoid
BPAG: bullous pemphigoid antigen
COPD: chronic obstructive pulmonary disease
DEJ: dermal-epidermal junction
DH: dermatitis herpetiformis
DPP-4i: dipeptidyl peptidase-4 inhibitor
DPPI: dipeptidyl peptidase I
enzyme-linked immunosorbent assay: ELISA
EBA: epidermolysis bullosa acquisita
Gzm: granzyme
human leukocyte antigen: HLA
Kitl: kit ligand
LABD: linear IgA bullous dermatosis
LAD-1: linear IgA disease antigen-1
LABD97: linear IgA bullous disease antigen of 97 kDa
LPP: lichen planus pemphigoid
mMCP-4: Mouse Mast Cell Protease 4
MMP: Matrix metalloproteinase
MMPh: mucous membrane pemphigoid
NE: neutrophil elastase
NK: natural killer
Pas: plasminogen activators
PAR-2: protease-activated receptor 2
PG: pemphigoid gestationis
PI-9: proteinase inhibitor-9
pmp: per million people
ROS: reactive oxygen species
Scf: stem cell factor
TIMP: tissue inhibitor of matrix metalloproteinase.
